# The use of fibroblasts as a valuable strategy for studying mitochondrial impairment in neurological disorders

**DOI:** 10.1186/s40035-022-00308-y

**Published:** 2022-07-04

**Authors:** Margrethe A. Olesen, Francisca Villavicencio-Tejo, Rodrigo A. Quintanilla

**Affiliations:** Laboratory of Neurodegenerative Diseases, Facultad de Ciencias de La Salud, Instituto de Ciencias Biomédicas, Universidad Autónoma de Chile, Santiago, Chile

**Keywords:** Mitochondria, Fibroblasts, Cell reprogramming, Alzheimer's disease, Parkinson’s disease, Huntington’s disease, Amyotrophic lateral sclerosis, Mitochondrial dysfunction

## Abstract

Neurological disorders (NDs) are characterized by progressive neuronal dysfunction leading to synaptic failure, cognitive impairment, and motor injury. Among these diseases, Alzheimer's disease (AD), Parkinson's disease (PD), Huntington’s disease (HD), and amyotrophic lateral sclerosis (ALS) have raised a significant research interest. These disorders present common neuropathological signs, including neuronal dysfunction, protein accumulation, oxidative damage, and mitochondrial abnormalities. In this context, mitochondrial impairment is characterized by a deficiency in ATP production, excessive production of reactive oxygen species, calcium dysregulation, mitochondrial transport failure, and mitochondrial dynamics deficiencies. These defects in mitochondrial health could compromise the synaptic process, leading to early cognitive dysfunction observed in these NDs. Interestingly, skin fibroblasts from AD, PD, HD, and ALS patients have been suggested as a useful strategy to investigate and detect early mitochondrial abnormalities in these NDs. In this context, fibroblasts are considered a viable model for studying neurodegenerative changes due to their metabolic and biochemical relationships with neurons. Also, studies of our group and others have shown impairment of mitochondrial bioenergetics in fibroblasts from patients diagnosed with sporadic and genetic forms of AD, PD, HD, and ALS. Interestingly, these mitochondrial abnormalities have been observed in the brain tissues of patients suffering from the same pathologies. Therefore, fibroblasts represent a novel strategy to study the genesis and progression of mitochondrial dysfunction in AD, PD, HD, and ALS. This review discusses recent evidence that proposes fibroblasts as a potential target to study mitochondrial bioenergetics impairment in neurological disorders and consequently to search for new biomarkers of neurodegeneration.

## Introduction

Alzheimer’s disease (AD), Parkinson’s disease (PD), Huntington’s disease (HD), and amyotrophic lateral sclerosis (ALS) are mainly characterized by neuronal dysfunction, oxidative damage, and mitochondrial impairment [1, 2]. Aging is the leading risk factor for either AD or PD; however, for HD and ALS, this process has not been considered a contributor to the onset of these diseases [3]. Due to the worldwide increase of the aging population, the occurrence of neurological disorders (NDs) has been rapidly expanded, leading to an increase in health economic cost [4]. Nowadays, it is widely accepted that NDs are multifactorial disorders, where mitochondrial dysfunction is considered a key hallmark in each of them [5–9]. Mitochondria are dynamic organelles that travel through the neuronal cytoskeleton in response to synaptic activity [10], contributing to energy supply, calcium regulation, and oxidative defences [11].

In AD, PD, HD and ALS, abnormalities of mitochondrial bioenergetics are characterized by reactive oxygen species (ROS) overproduction, ATP deficiency, reduced oxidative phosphorylation (OXPHOS), and calcium mishandling. However, abnormalities in mitochondrial dynamics regulation and mitophagy could also affect these organelle bioenergetics properties [9, 12–17].

Accumulative studies using neuronal cells, animal models, and patient samples have shown that the early cognitive deficiencies correlate with mitochondrial bioenergetics failure in AD, PD, HD, and ALS [12, 18–24]. In this context, studies in skin fibroblasts obtained from patients with these diseases have generated a broad impact on research because of their ability to mimic early mitochondrial bioenergetics defects observed in these disorders [25–29]. Furthermore, skin fibroblasts are peripheral cells that can be accessed non-invasively from patients, and they present the capacity to be efficiently reprogrammed into neuronal cells [27, 30]. Fibroblasts are considered as an exciting model for studying NDs like AD, PD, HD and ALS since they reproduce the metabolic changes observed in neurons [31–35]. More importantly, recent findings have reported that skin fibroblasts show oxidative damage, reduced ATP levels, and mitochondrial fission abnormalities, which correlate with the features observed in ND patient samples [36, 37].

**Fig. 1:**
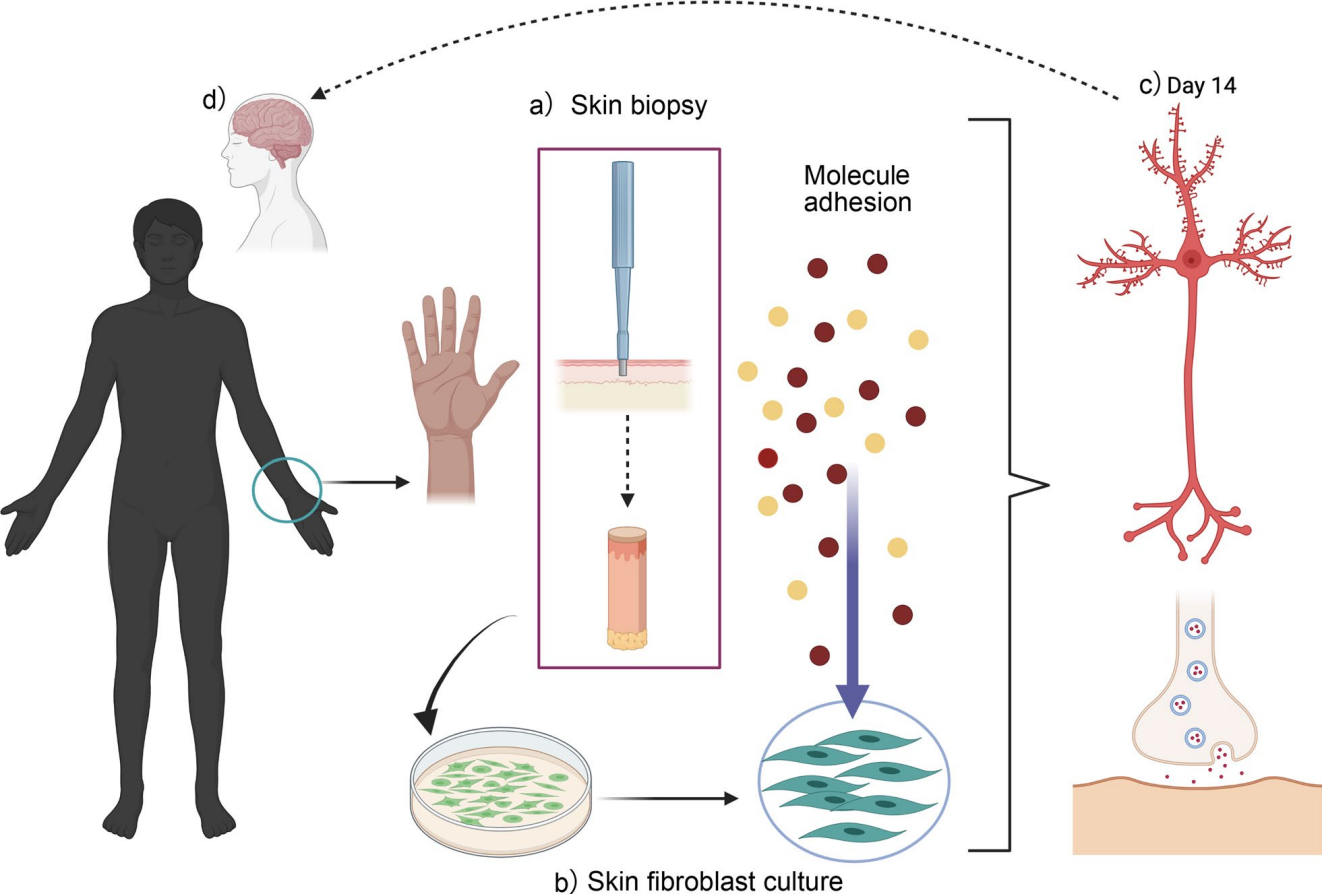
Effective conversion from human skin fibroblasts into neurons. Studies have determined an effective protocol for reprogramming human skin fibroblasts into neurons. First, fibroblasts are collected by skin biopsy from a human patient (**a**). Then, fibroblasts are cultured in a cell growth medium supplemented with chemical molecules inducing reprogramming into different types of neurons (**b**), such as glutamatergic, dopaminergic, GABAergic, and motor neurons. Furthermore, reports have suggested that this conversion is successfully evident at day 14, when neuronal features are observed, such as the presence of prolongations (dendrites and axons) and neuronal soma, along with physiological neuronal features (**c**) that represent brain function (**d**)

Overall, fibroblasts have been widely used for mitochondrial analysis in NDs, offering a better approach to understanding the onset and progression of NDs [38–41]. Therefore, this review will discuss evidence for the patient-derived skin fibroblasts as an exciting tool to study abnormalities of mitochondrial bioenergetics in AD, PD, HD and ALS (Fig. 1).

## Mitochondrial dysfunction in AD, PD, HD and ALS

### Overview of mitochondrial function

Mitochondria are organelles delimited by the outer membrane (OMM) and inner membrane (IMM) [42]. OMM includes a voltage-dependent anion-selective channel (VDAC), which allows the flow of small molecules (5 kD) from the cytoplasm toward mitochondria [42]. While the IMM contains five complexes of the electron transport chain (ETC) where OXPHOS takes place, both membranes give rise to the intermembrane space and the mitochondrial matrix is where the tricarboxylic cycle occurs [42]. Mitochondria maintain a dynamic behaviour through the balanced processes of fusion and fission (mitochondrial dynamics), which allows the maintenance and exchange of mitochondrial content and the removal of its damage (mitophagy) [43]. The main specialized proteins controlling OMM fusion are Mitofusin 1 and 2 (Mfn1/2), while for the IMM, it is optic atrophy protein 1 (OPA1); in addition, the critical protein for mitochondrial fission is Dynamin-related protein 1 (DRP1) which is recruited from the cytosol towards mitochondria by mitochondrial adapter protein Fis1 [43]. A regulated fusion/fission process is strongly associated with a correct mitochondrial function, while its dysregulation could lead to mitochondrial dysfunction and neuronal death [44, 45].

Cellular energy provided by mitochondria is obtained through ATP synthesis within the mitochondrial matrix via the ETC complexes I, II and III, which pump protons into the mitochondrial intermembrane space to finally activate the catalytic activity of ATP synthase (complex V), generating the ATP synthesis [46]. This protein complex drives a mechanical rotation inducing conformational changes in the nucleotide-binding pockets of the F1 subcomplex, leading to the conversion of ADP and phosphate to ATP [46]. ETC is composed of complex I (NADH-ubiquinone oxidoreductase) which initiates the entry of electrons in the ETC, complex III (ubiquinone-cytochrome *c* oxidoreductase) that couples the electron transfer from QH_2_ to cytochrome-translocating protons across the IMM [47], and complex IV (cytochrome *c* oxidase) which is the terminal oxidase in the ETC and reduces O_2_ to H_2_O [47]. The complexes I and III are the main producers of ROS, which are considered as secondary products of ETC activity. The complex II (succinate dehydrogenase) does not pump protons across the IMM but catalyzes the oxidation of succinate into fumarate, together with the reduction of ubiquinone to ubiquinol [46]. Importantly, besides NADH, ubiquinol is also a substrate for complex III, providing another connection between the citric acid and the ETC [48–50].

Under normal conditions, mitochondrial ROS production is balanced by antioxidant defences and plays physiological roles in cell signalling; however, ROS overproduction could trigger a cascade of abnormal effects, including mitochondrial failure and oxidative damage [51, 52]. In addition, mitochondria contribute to cell calcium regulation by the action of mitochondrial calcium uniporter, mitochondrial permeability transition pore (mPTP), and Na^+^/Ca^2+^ and H^+^/Ca^2+^ exchangers [53–55]. Calcium regulation is vital for exocytosis, neurotransmitter vesicle recycling, and communication between neurons [56–58]. However, mitochondrial calcium defects accompanied by oxidative damage and ATP deficit could lead to neuronal dysfunction [59–61]. Given the high energetic demand of the brain, which accounts for 20% of resting-state oxygen consumption [62], it is expected that abnormal mitochondrial functions may be involved in the pathogenesis of main NDs and its improvement could potentially ameliorate neurodegeneration in AD, PD, HD and ALS. In this context, emerging evidence has demonstrated that the nuclear factor (erythroid-derived 2)-like 2 (Nrf2) plays a significant role in the antioxidant response promoting neuroprotection related to mitochondrial health [62, 63]. In particular, our group and others have demonstrated that activation of the Nrf2 pathway prevents mitochondrial dysfunction in AD models [64–66] and PD models [67, 68].

### AD

AD is the most common neurodegenerative disease, with a prevalence of ~ 24 million people worldwide among the elderly over 60 years of age [69]. AD is clinically characterized by memory impairment, cognitive deficits, abnormalities in speech, and spatial disorientation [70]. The AD brain is characterized by accumulation of extracellular plaques (senile plaques) formed by aggregates of beta-amyloid (Aβ) peptide (1–40; 1–42) and neurofibrillary tangles (NFTs) formed by aberrantly hyperphosphorylated, truncated, and other pathological forms of tau protein [71]. Mutations in the amyloid protein precursor (APP) or in the catalytic subunits of γ-secretase presenilin-1 (PSEN1) and presenilin-2 (PSEN2) are related to familial cases of AD (fAD) with an early onset of the disease, which represents the 5% of AD cases [72]. Sporadic AD (sAD), which represents the majority of AD cases, has an unknown etiology [72]. An increasing number of studies have demonstrated that the mitochondrial damage could be a potential biomarker to track the progression of sAD [16, 73, 74]. In this context, defects in mitochondrial dynamics have been observed in AD, leading to abnormal mitochondrial distribution and synaptic failure [75–77]. Furthermore, key proteins for the regulation of mitochondrial dynamics have been evaluated in the post-mortem brains of AD patients [76]. These studies showed abnormal expression of DRP1 (a 74.3% decrease), Mfn2 (33.6% decrease), Mfn1 (27.8% decrease) and OPA1 (61.4% decrease). On the contrary, Fis1 expression is increased 4.8-fold in AD brains, which is associated with mitochondrial accumulation in the neuronal soma [76].

**Fig. 2:**
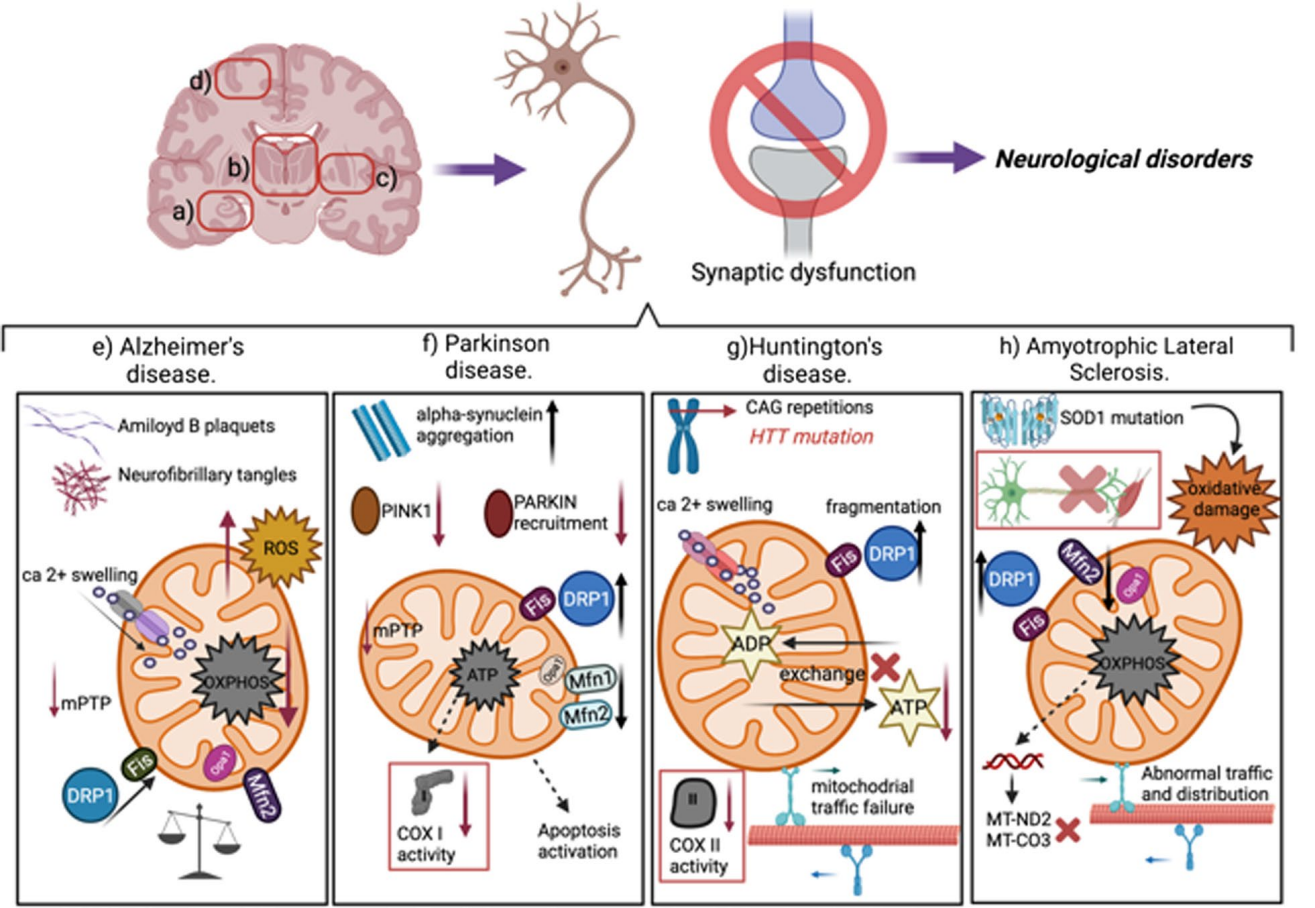
Mitochondrial dysfunction is a critical hallmark of neuronal failure present in AD, PD, HD, and ALS. Neuronal dysfunction is considered to be an early and pathological event in NDs. Hippocampal neuronal dysfunction in AD (**a**), neuronal failure in substantia nigra in PD (**b**), neuronal dysfunction in basal ganglia and striatum in HD (**c**), and motor cortex damage in ALS (**d**). Evidence has strongly suggested that mitochondrial failure plays a crucial role in neuronal dysfunction observed in NDs. In AD, bioenergetic failure is observed, which includes ROS overproduction, reduced ATP production, mitochondrial depolarization, calcium handling defects, along with excessive mitochondrial fragmentation (**e**). In PD, mitochondrial dysfunction is characterized by mitophagy failure. Several reports indicate that loss of PINK1 and Parkin leads to neuronal dysfunction. Also, mitochondria show a reduction of mitochondrial complex I activity, decreased Δψm, and increased mitochondrial fragmentation (**f**). In HD (**g**) and ALS (**h**), mitochondrial bioenergetic dysfunction is also reported, along with mitochondria traffic failure in neurites, preventing the arrival of mitochondria to areas of high energy demand, therefore leading to synaptic failure

Also, complementary studies showed that the interaction of Aβ with DRP1 leads to the decrease of mRNA expression of mitochondrial fusion genes Mfn2, Mfn1, and OPA1 in AD brain samples (Fig. 2) [78]. However, mRNA expression of mitochondrial fission genes (DRP1 and Fis1) was increased at early stages of the disease [78]. Importantly, Chang and co-workers using a PCR-based technique, showed an increase in the frequency of point mutations in mitochondrial DNA (mtDNA) in the parietal gyrus, hippocampus, and cerebellum of AD brains compared to age-matched controls [79]. Furthermore, complementary studies in sAD patients showed a significant reduction of complexes I and IV activities in the cortex and hippocampus [80, 81]. Also, reduced mRNA expression of glycolysis, TCA cycle, and OXPHOS proteins has been shown in the hippocampus of post-mortem AD brains [82].

Importantly, additional studies have suggested an essential role of pathological forms of tau in mitochondrial failure in AD [83–85]. For example, caspase-3-cleaved tau (truncated tau) is a pathological form present in the early stages of AD and accumulating in NFTs [86]. In this context, expression of this truncated tau in primary neurons and immortalized cortical neurons reduces the expression of TRAK2 (mitochondrial motor protein) and increases the mitochondria/TRAK2 binding, leading to impairment of mitochondrial transport [87]. Also, the expression of caspase-3-cleaved tau induces mitochondrial fragmentation by reducing the expression of Mfn2 and Opa1 [85]. In addition, the presence of abnormal hyperphosphorylation of tau and tau-P301L are also suggested to induce mitochondrial failure in AD [88, 89].

Importantly, pathological hyperphosphorylation of tau promotes its aggregation, leading to the development of AD [90]. It has been indicated that the presence of tau hyperphosphorylated at Ser396/Ser404 sites (epitope known as PHF-1) induces mitochondrial dysfunction [91]. Moreover, studies in neuronal cybrid cells obtained by transplanting platelet mitochondria from AD patients into human neuroblastoma cells (SH-5YSY) showed decreased mitochondrial length and density along with exacerbated mitochondrial fragmentation [92].

Calcium dysregulation has also been described as an essential contributor to the pathogenesis of AD [93, 94]. In this regard, defects in mitochondrial calcium have been associated with AD progression [93]. For instance, it has been observed that neuronal excitotoxicity provoked by excessive stimulation with excitatory glutamate, induces massive calcium influx in the in vitro AD model [95, 96]. In this context, other studies indicate that the glutamate receptor, *N*-methyl-*D*-aspartate receptor (NMDAR), plays a predominant role in the excitotoxicity process since it allows the massive calcium influx in neurons. Nevertheless, selective NMDAR blockade by antagonist MK-801 reduces neuronal toxicity in cultured rat hippocampal neurons [97]. Furthermore, mitochondrial dysfunction has been suggested as a contributing factor to glutamate neurotoxicity since mitochondria participate in calcium regulation [98]. In addition, evidence has shown that the mitochondrial failure precedes neurotoxic events induced by glutamate, by reducing ATP synthesis and Δψm (mitochondrial membrane potential) in hippocampal rat neurons [99]. More importantly, blocking of mPTP (mitochondrial calcium regulator) by cyclosporin A (an inhibitor of mPTP opening) prevents mitochondrial depolarization and excitotoxicity induced by NMDAR stimulation in the hippocampus [99].

Furthermore, studies in P301L mutant mice (a mouse model that shows accumulation of hyperphosphorylated tau and NFT formation) showed that the mitochondrial function is negatively affected by decreased expression of the complex V and decreased complex I activity [89]. Also, the mitochondrial respiratory capacity is impaired, shown as reductions in respiratory state 3 and ATP synthesis [89]. Interestingly, using the same mouse model, Teresa et al. showed a 50% reduction in mitochondrial number per μm in axons, deficient mitochondrial transport, and mitochondrial clustering in somatodendritic zones [100]. Finally, other studies in the P301L mice suggested that tau phosphorylated at the PHF-1 site is responsible for the induction of mitochondrial fragmentation, ROS increase, lipid peroxidation, reduced cytochrome *c* activity, and ATP loss, which negatively impact cognitive performance [101]. Defects of mitochondrial dynamics have been observed in the hippocampus of APP transgenic mice, which display reduced mitochondrial elongation and increased fragmentation at three months, and abnormal mitochondrial distribution was also observed, as confirmed by the reduced mitochondrial population in synaptic buttons [77].

In AD, defects in mitochondrial bioenergetics have been observed [16, 20, 102–104]. For example, reduced expression of OSCP (F1F0-ATP synthase subunit) has been found in 5× FAD mice (a mouse model that expresses human APP and PSEN1 transgenes with five AD-linked mutations: K670N/M671L, I716V and V717I mutations in *APP*, and L286V and M146L mutations in *PSEN1*), which negatively affects mitochondrial and synaptic functions [105]. In addition, the hippocampus of 3× Tg-AD mice (a mouse model that expresses three mutations associated with AD: APP, P301L, and PSEN1) exhibits age-related decrease of expression of mitochondrial complex IV with increased production of H_2_O_2_ and lipid peroxidation [106].

Furthermore, ATP bioluminescence analysis showed a time-dependent reduction of ATP in the APP mouse brains [102]. The Δψm loss is another sign of mitochondrial dysfunction in AD [107]. For example, the APP transgenic mice show decreased Δψm in the cortex compared to age-matched control animals [108].

In summary, these studies highlight three critical aspects of mitochondrial function that are compromised in AD: (1) mitochondrial dynamics, (2) bioenergetics (ATP, ROS production, calcium regulation), and (3) mitochondrial transport (synaptic function) (Fig. 2).

### PD

PD is the second most common neuropathological disorder after AD, with an incidence of 4.1 million people per year, being more common in the male population with a predicted increase of 8.7 million affected per year in 2030 [109]. The clinical symptoms of PD include bradykinesia, tremor, rigidity, and postural and gait instability, disabling the person as the disease progresses [110]. PD is characterized by dopaminergic neuron loss in the substantia nigra pars compacta (SNpc), which results in the loss of dopaminergic inputs in the striatum and motor abnormalities present with the disease [111–113]. Accumulation of α-synuclein aggregates in Lewy bodies in the SNpc is the pathological hallmark of PD [22, 114]. It was previously considered that sporadic cases were the primary origin of this disease [115]. Currently, it is widely accepted that environmental and genetic factors also contribute to the onset of PD [115–117]. Notably, over > 90% of PD cases correspond apparently to sporadic PD patients (sPD), lacking a family history [118]. However, recent data suggest that sPD can also have a genetic basis, although more research is needed [119]. Otherwise, the familial PD (fPD), which includes monogenic forms of PD that are usually referred to as fPD, is related to genetic mutations that affect cellular pathways central to the PD pathophysiology [118, 120]. Significantly, most genetic PD loci are directly associated with mitochondria; therefore, mitochondrial impairment has been implicated as an essential feature of PD [121].

Accumulating evidence indicates that neurodegenerative changes in PD are associated with mitochondrial dysfunction in the SNpc and the frontal cortex, including impairment of mitochondrial biogenesis, increased ROS production, mitochondrial fragmentation, and Ca^2+^ deregulation [122–126] (Fig. 2). Neuropathological studies showed reduced expression of mitochondrial complex I in the striatum and substantia nigra (SN) of PD patients, although in these studies, the type of PD was not specified [127, 128]. Also, studies in animal models based on chronic administration of 1-methyl-4-phenyl-1,2,3,6-tetrahydropyridine, rotenone or paraquat, can reproduce the neurodegenerative features observed in PD [129–131]. Interestingly, these effects are produced from the inhibitory effects of these compounds on mitochondrial complex I, corroborating evidence from PD patients [129–131]. Consistently, another study that evaluated respiratory chain activity in single neurons from sPD patients showed alterations in complexes I and II [132].

Abnormalities in mtDNA have been observed in different studies related to the pathogenesis of PD [132, 133]. For example, a reduced neuronal mtDNA copy number was found in PD patients compared to age-matched patients [133]. Furthermore, these changes in mtDNA are accompanied by an accumulation of deleted mtDNA in PD patients, which could increase the levels of somatic mutations, contributing to mitochondrial bioenergetic defects observed in PD neurons in the SNpc [133].

Related to mtDNA defects, it is essential to discuss mutational changes in PINK1 and Parkin proteins and their contribution to mitochondrial dysfunction present in fPD. Mutations in the PINK1 (*PARK6*) and parkin (*PARK2*) genes are related to the autosomal recessive early-onset fPD [134, 135]. Both Parkin and PINK1 participate in the mitophagy process through the Parkin-mitochondria association induced by PINK1 activity [136]. Mitophagy is the process that removes damaged mitochondria to maintain adequate quality control [137]. Parkin contributes to mitophagy and regulates the selection of damaged organelles [138]. Mitophagy starts with the binding of PINK1 with Parkin [138], which induces the phosphorylation of Parkin, resulting in their translocation to mitochondria to initiate the ubiquitination process of mitochondrial substrates. Mutations or loss of PINK and Parkin has been linked to PD [139, 140]. Studies have shown that the presence of mutant PINK1 or Parkin in PD patients inhibits the mitophagy process, leading to accumulation of impaired mitochondria, and consequently neuronal damage typical of PD [139, 140].

Also, it has been observed that the PINK1 (-/-) or Parkin (-/-) deficient mice show oxidative damage, which is enhanced as age increases [141, 142]. Importantly, these changes are also observed in a Pink1-deficient *Drosophila* model of PD, leading to synaptic deficiency [142, 143]. Furthermore, either a loss of or deficiency in PINK1 and Parkin expression could decrease the mitochondrial energy production, impair the complex I respiratory activity, induce mitochondrial depolarization, and increase apoptotic activity [143, 144].

Another relevant mutation in PD is the mutation of leucine-rich repeat kinase 2 (LRRK2), which provokes an autosomal dominant form of PD and has been identified as the most common cause and a genetic risk factor for fPD [145]. LRRK2 is a multifunctional protein kinase whose increased kinase activity is related to its pathological activity [146]. Importantly, it has been observed that LRRK2 also contributes to the risk of sPD [147].  A majority of LRRK2 carriers have clinical and pathological features indistinguishable from sPD, indicating a critical role of LRRK2 in PD pathogenesis [148]. The PD-associated mutation G2019S (kinase domain) is thought to increase the kinase activity of LRRK2 [149]. Normal levels of the most commonly known LRRK2 G2019S mutant have been observed with mitochondrial abnormalities in patient-derived dopaminergic neurons [150] and LRRK2 G2019S knock-in mice [151].

Another critical feature of PD is the abnormal mitochondrial dynamics caused by increased DRP1 expression [152, 153]. In particular, in the NT2 derived cybrid cells obtained from tetracarcinoma cells and platelet mitochondria from sPD patients, mtDNA depletion by long-term bromide ethidium exposure results in reduced expression of complex I [153]. These abnormalities are accompanied by an increase in mitochondrial fission due to the cleavage of OPA1 long isoform and the increase of DRP1 phosphorylation levels [153].

In addition, the Mfn1/Mfn2 expression is found to be decreased in PD [154], affecting neuronal function and locomotor activity in PD mouse model [155]. This evidence suggests that the increased DRP1-dependent mitochondrial fragmentation can mediate toxicity and impair mitochondrial distribution in dopaminergic neurons, thus contributing to the pathogenesis of PD [156]. Also, other studies have suggested that these defects of mitochondrial dynamics could contribute to or be associated with the pathological aggregation of α-synuclein in PD [154, 157]. Importantly, in vitro evidence has shown significant mitochondrial fragmentation in Hela cells co-transfected with human protein α-synuclein [158].

Therefore, mitochondrial dysfunction plays an integral role in the pathogenesis of sporadic and familial PD and has a consequential impact on neurodegeneration.

### HD

HD is a dominantly inherited disease caused by the repetition of CAG (cytosine, adenine, and guanine) trinucleotides of the huntingtin (HTT) protein gene located on chromosome 4 [159, 160]. The clinical progression of HD is divided into periods of pre-manifestation (10–15 years before symptom onset) and manifestation (symptoms present) [159]. The clinical symptoms of HD are mainly motor (involuntary movement), cognitive and psychiatric dysfunctions leading to dependence on daily life, and finally death [161]. HD prevalence is 2.71 per 100,000 people, and this number is even higher in Europe, North America, and Australia, which present a prevalence of 5.7 per 100,000 habitants. Overall, HTT protein has a critical role in the development of the nervous system and neuronal survival [162, 163]. More importantly, HTT mutation leads to mitochondrial failure and neuronal dysfunction [164, 165]. Therefore, mitochondrial dysfunction likely plays a crucial role in the pathogenesis of HD [166]. Mitochondrial function alterations observed in animals and patients of HD include altered Ca^2+^ regulation, ROS increase, excitotoxicity, and defects in mitochondrial dynamics and trafficking [167]. Also, it has been demonstrated that an increase in mitochondrial ROS production and mtDNA lesions could compromise the mitochondrial respiratory capacity in striatal cells that express mutant HTT (mHTT) [167].

Also, other studies showed that mHTT expression reduces the activity of mitochondrial complex IV along with mitochondrial depolarization in lymphoblasts of human patients and primary neuronal cultures [17, 168]. Importantly, the ST HdhQ111 striatal cells, a clonal striatal cell line that expresses mutant HTT with 111 CAG repeats, show a decreased production of mitochondrial ATP and lower mitochondrial ADP-uptake. This metabolic blockage is associated with enhanced Ca^2+^ influx through NMDARs, which, when they were blocked, result in decreased ATP/ADP ratio in cells with expanded CAG repeats [168].

Additionally, studies from Milakovic and colleagues reported that mitochondria are more susceptible to calcium stress in clonal striatal cells that express mutant HTT (111 CAG) HD [169]. Also, other reports demonstrated that the SH-SY5Y human neuroblastoma cells that express mutant HTT present a significant association of this protein with mitochondrial fraction (specifically in OMM), leading to calcium dysregulation, cytochrome *c* release, swelling, and mPTP opening [170]. Interestingly, previous findings from our group showed that striatal cells expressing mutant HTT protein (STHdh^Q111/Q111^) and cortical neurons expressing mutant-HTT protein show mitochondrial dysfunction (depolarization, fragmentation and calcium dysregulation) in response to thapsigargin (a SERCA antagonist that induces a transient cytosolic calcium increase) treatment [164, 165]. Interestingly, these mitochondrial abnormalities are prevented by cyclosporine A, which is a specific inhibitor of mPTP opening [164, 165]. These observations suggest a potential role of mPTP in the mitochondrial dysfunction induced by calcium stress in mutant huntingtin cells [164–166].

Mitochondrial dynamics have been documented to be significantly affected in HD [60]. Mitochondria in HD show dynamic imbalance with increased DRP1 and Fis1 and reduced Mfn1/2 and OPA1 expression, leading to a decrease in mitochondrial number and neuronal atrophy in HD [60, 171], as well as impaired mitochondrial axonal transport [164]. However, treatment with peptide P110 (suppressor of DRP1 activity) in the zQ175 knock-in mouse model (zQ175 allele encodes the human HTT exon one sequence with a ~ 190 CAG repeats) disables the translocation of DRP1 into mitochondria, improves locomotor activity and reduces the white matter degeneration of the corpus callosum in HD mice [172]. Furthermore, mutant HTT and its relationship with mitochondria have been studied in the brains of Hdh (CAG)150 knock-in mice (HD mouse model that carries ~ 150 CAG repeats in HTT locus), observing that the mutant HTT interacts with mitochondria and consequently affects anterograde and retrograde mitochondrial traffic [173]. Complementary findings have been found in the brains of HD patients by histopathology and western blot assays where DRP1 showed a progressive increase in the caudate nucleus, while Mfn1 was significantly reduced in the caudate nucleus of HD patients [60].

Also, the defective activity of OXPHOS has been described in HD. For example, activities of complexes II and III are decreased, leading to neuronal degeneration in the striatum of HD post-mortem brains [174, 175] (Fig. 2). Furthermore, biochemical analysis has revealed defective activities of the respiratory chain complexes and tricarboxylic acid (TCA) cycle enzymes in brains of HD patients. Specifically, these studies showed reduced activities of complexes II, III, and IV in the caudate and putamen nucleus of HD patients with advanced stages of the disease [176–178]. Overall, the above evidence suggests that mitochondrial dysfunction is crucial for neuronal susceptibility in HD [60].

### ALS

ALS is a neurodegenerative disease that affects motor neurons and clinically presents progressive muscular atrophy with generalized fasciculation, spasticity, dysarthria, dysphagia, and dyspnea, with increased severity as the disease progresses [179]. ALS has a prevalence of approximately 6 cases per 100,000, and patients present survival times of 3–4 years [180].

Mitochondrial dysfunction has also been involved in ALS apparently caused by the presence of superoxide dismutase 1 (SOD1) mutations which alter mitochondrial metabolism and induce apoptosis of motor neurons [181, 182] (Fig. 2). Furthermore, the ALS transgenic animal model based on mutant SOD1 expression shows disrupted mitochondrial motility, affecting axonal mitochondria [183, 184]. Mitochondrial bioenergetics can also be affected in ALS [186]. According to this, examination of the ventral horn and the spinal motor neurons of ALS patients showed a significant decrease in *MT-ND2* (Complex I) and *MT-CO3* (Complex IV) genes, which could lead to the inhibition of ATP synthesis and motor neuronal degeneration [186–188]. Importantly, impairment of mitochondrial dynamics has been found in ALS, generating motor neuron toxicity and degeneration [19, 185, 189, 190]. For example, in cultured motor neurons from mutant SOD1 transgenic mice, mitochondria are less interconnected and significantly fragmented [189]. In addition, axonal mitochondria were observed to be minor and with an altered distribution, suggesting that this event occurs prior to clinical symptoms [189]. These alterations in mitochondrial localization could be caused by mitochondrial fragmentation caused by an increase in DRP1 expression affecting motor neuron viability [191–193] (Fig. 2).

Furthermore, analysis of mitochondrial morphology in the spinal cords of ALS patients showed evident disorganization of cristae and accumulation of mitochondria in the neuronal soma, which indicate an abnormality in mitochondrial transportation [194]. Inhibition of mitochondrial fragmentation by intraperitoneal Mdivi-1 (DRP1 inhibitor) reverses the mitochondrial morphological alterations in the mutSOD1-G93A transgenic mouse model of ALS [192]. Complementary studies in this model showed mitochondrial deficiencies caused by the interaction of Bcl-2 protein with VDAC, affecting the permeability of the OMM and inhibiting ADP transport, leading to ATP deficiency and neuronal apoptosis [195]. Also, another study in mutSOD1-G93A transgenic mice showed reduced mitochondrial respiration in state 3 (after ADP was added) in the liver, brain, and spinal cord [196]. Also, a significant reduction in activities of mitochondrial respiratory chain complexes I-IV in the spinal cord of mutant SOD1 mice was observed [196]. Furthermore, mitochondrial calcium handling defects have been detected in ALS, mainly in muscle fibers where axonal degeneration occurs in ALS [197].

Interestingly, studies in cultured primary motor neurons overexpressing TDP-43 showed reduced mitochondrial length and density in neurites of primary motor neurons, and these features were highly exacerbated by ALS-associated TDP-43 mutants Q331K and M337V [198]. On the contrary, the authors also indicate that suppressing TDP-43 expression resulted in significantly increased mitochondrial length and density in neurites, indicating a specific role of TDP-43 in regulating mitochondrial dynamics [198]. TDP-43 is a protein that contributes to ALS pathophysiology [198]. In normal conditions, TDP-43 is a protein of 414 amino acids found in the cell nucleus and encoded by the TARDBP gene on human chromosome 1p36.2, which has been involved in the regulation of neuronal plasticity [198]. Furthermore, the impairment of mitochondrial axonal transport was also observed with a reduced mitochondrial anterograde and retrograde movement in motor neurons transfected with TDP-43 [198]. Similarly, motor neurons transfected with TDP-43 show a decrease in Mfn2 expression, and the induction of Mfn2 protein expression prevents abnormalities in mitochondrial dynamics and bioenergetics shown in this neuronal model of ALS [198]. Oxidative damage is considered one of the main contributors to the pathogenesis of NDs, and it has been observed in motor neurons of ALS patients [199–201]. In this context, the expression of mutant SOD1 in a human neuroblastoma cell line (SH-SY5Y) induces a decrease in OPA1 expression, probably due to the oxidative damage induced by the presence of mutant SOD1 and the increase of DRP1 (P-DRP1 s616) [202].

Altogether, this evidence indicates that mitochondrial dysfunction has a pivotal role in the onset and progression of AD, PD, HD, and ALS. Unfortunately, techniques or models that facilitate identification of mitochondrial defects at early stages of these diseases have not been fully identified. However, the use of skin fibroblasts from AD, PD, HD, and ALS patients provides a new tool to study the role of mitochondrial dysfunction in the pathogenesis of these diseases.

## Skin fibroblasts: a model to study pathological changes in AD, PD, HD, and ALS

In recent years, there has been an urgent need to develop novel methodologies to understand how different inherited and sporadic NDs can compromise mitochondrial function and neuronal survival [116, 170, 203]. Considering that post-mortem analyses cannot provide precise information on disease progression, it is fundamental to develop new model systems that enable investigations of early molecular changes, which may be relevant as targets for novel therapeutic options.

In particular, the use of skin fibroblasts represents an exceptional complementary tool for investigating these neurodegenerative processes [38, 39, 41, 204]. These cells can recapitulate early pathological events shown in the brains of AD, PD, HD, and ALS patients [38, 39, 41, 204]. One of the essential strategies to study neurodegeneration in fibroblasts obtained from ND patients is the differentiation of these cells into proper functional neurons [205–209]. This wide range of protocols for fibroblast differentiation represents a relevant source of knowledge, providing results and phenotypes closer to the human patients suffering from NDs.

In this context, in the following two sections, we summarize primary literature concerning the methodologies to facilitate fibroblast differentiation into neurons to study the pathogeneses of AD, PD, HD, and ALS. Also, we will briefly discuss the production of induced pluripotent stem cells (iPSCs) and the direct reprogramming method as a valuable tool to convert fibroblasts into neurons and to study defects of mitochondrial bioenergetics in NDs.

### iPSCs

Cellular reprogramming encompasses many techniques that enable researchers to reverse or take possession of the differentiation path of mature cells [205]. For example, iPSCs and direct reprogramming, also named trans-differentiation, have partially solved the limited availability of primary cells in patients. Also, the use of iPSCs has helped in various studies on the recapitulation of physiological and pathological mechanisms in patient-derived lines [206–208]. In addition, significant advances in gene-editing technologies—particularly the CRISPR/Cas9 technology—enable the fast generation of genetically defined human hiPSC-based disease models [210]. This technology can be helpful in patient-derived iPSCs with AD, PD, HD or ALS phenotype, providing additional models to study the pathological mechanisms involved in these diseases [211–213].

Indeed, patient-derived iPSC models have been generated and validated, especially for their capacity to progressively express the primary pathological markers, such as aggregation of Aβ, tau hyperphosphorylation, and α-synuclein misfolding, which are typical hallmarks observed in tissues from AD and PD patients [214–216]. Importantly, sAD-derived neurons or non-AD-derived neurons treated with Aβ present standard features like mtDNA damage, mitochondrial dysfunction, and increased ROS production [217].

In this context, the iPSC models are a powerful tool for exploring APP processing in tissue-specific cells from individuals with fAD-causing mutations [218]. Other studies have shown increased levels of secreted Aβ42 in neurons with the PSEN1 A246E and PSEN2 N141I mutations [219]. For instance, a study on neurons bearing pathogenic PSEN1 mutations showed an increased ratio of Aβ42:40 [220]. Moreover, Israel et al. modeled both fAD and sAD phenotypes and found increased levels of Aβ40 and tau phosphorylation in the iPSC-derived neurons of fAD and sAD patients compared with neurons from non-demented age-matched individuals [221].

Many studies based on iPSC have reported mitochondrial defects in PD-derived neurons and defined the underlying mechanisms. For example, expression of mutant LRRK2 and overexpression of wild-type LRRK2 in patient-derived iPSCs induce mitochondrial fragmentation that leads to ROS production, resulting in increased vulnerability to cell stressors [222]. In addition, iPSC-derived dopaminergic neurons generated from R1441C mutation carriers exhibit increased oxidative stress, cell death, and impaired neuronal differentiation [222]. These studies showed morphological and functional changes of mitochondria in PD patient iPSC-derived neurons [222, 223]. Moreover, decreased levels of adenine dinucleotide (NAD+), a vital metabolic substrate involved in cellular energy production, are shown in PD iPSC-derived DA neurons [223]. It is important to mention that cell populations generated from the current protocols remain heterogeneous and may contain various percentages of non-DA neurons, such as motoneurons and GABAergic neurons, neural progenitor cells and undifferentiated cells [224].

Several groups have successfully generated specific HD-iPSCs that can be differentiated into HD neural stem cells (HD-NSC) and neurons with striatal features [225–228]. For example, Lopes et al. observed in early differentiated human HD-iPSC and NSC key features of mitochondrial and metabolic impairment related to decreased activities of complex III and pyruvate dehydrogenase complex (PDH), decreased oxygen consumption and mitochondrial ATP production, excess ROS production and mitochondrial fragmentation [229]. Furthermore, other studies found decreased ATP and glycolytic enzyme expression in HD iPSC-derived striatal neurons, although preserved or upregulated mitochondrial-related mRNA and protein expression was observed [230].

In ALS, it is still challenging to model the two main phenotypic alterations, motor neuron death and axon degeneration, in iPSC-derived cultures. First, the efficiency of motor neuron generation is extendedly variable between protocols and from one iPSC clone to another [231, 232]. Second, the motor neuronal cell bodies tend to form enormous clusters that are inaccessible for cell survival [233–235]. Finally, motor neuron axons usually display extensive crisscrossing and tangling with axons of other types of neurons, thus making it difficult to identify and analyze the axons [231, 236]. Although related to mitochondrial bioenergetics, other studies showed a reduction in mitochondrial respiration, and an increase in glycolytic flux was reported in a cell model of ALS [237].

Previous evidence indicates the successful use of iPSCs to replicate neurodegenerative features and mitochondrial dysfunction in AD, PD, HD, and ALS. However, some concerns are raised related to the low efficiency of iPSC conversion and the increased risk of tumorigenesis, which is inherent when working with oncogenes and proliferating pluripotent cells (Table 1) [238]. In addition, the process of iPSC differentiation into functional neural cells is complicated and time-consuming that is still under scrutiny [239]. Briefly, the iPSC-derived neurons transit through an embryo-like stage; thus, the epigenetic codes induced by aging or the disease per se could be altered or even erased in the cells obtained by this process [240]. On the contrary, Xu and others have shown the switch of the phenotype of one somatic cell type to another in the process of cellular reprogramming, which is also called trans-differentiation, during which neurons or glial cells can be directly induced from somatic cells without the need of a stem cell-like stage [241, 242]. Moreover, the direct trans-differentiation with minor induction steps could be more efficient and safer for cell therapeutic application, so the tumorigenicity process involving iPSC could be potentially prevented [243].

**Table 1:** Comparison of fibroblast conversion into neurons using different methodologies

	Indirect conversion into neurons via hiPSC	Direct conversion into functional neurons
Advantages	Personalized use in patients Drug design and treatment Pluripotent Useful for studying pathological mechanisms Extended use in studies of NDs	Rapid and efficient differentiation and maturation Personalized use in patients Avoid gene materials Age-match Useful for studying aging and NDs Physical factors can work in vivo to directly reprogram fibroblasts
Disadvantages	Slow process of cell formation Rejuvenated differentiated cells are not helpful for studies of aging or age-related diseases Abnormal gene expression and epigenetic abnormalities Tumorigenic pluripotent cell stages	Low efficiency Limited use in clinical therapy because of neuronal culture issues Standardization of protocol is still under investigation Chemically induced neurons are mostly glutamatergic neurons

### Direct reprogramming of human fibroblasts to neuronal cells to study mitochondrial dysfunction in AD, PD, HD and ALS

Current studies have proposed the use of skin fibroblasts as a tool to study the genesis and progression of AD, PD, HD, and ALS [208, 244, 245]. Fibroblasts are spindle cells of mesenchymal origin found in connective tissues of the body [246, 247]. Fibroblasts are metabolically active peripheral cells with functions such as the production, secretion, synthesis, and homeostatic maintenance of extracellular matrix components such as collagen, fibrin, and proteoglycans in different tissues [246]. Fibroblasts are heterogeneous, plastic, and dynamic since they can differentiate according to their location and function in different tissues [248] (Fig. 1). In addition, fibroblasts can alter their physiology and be transformed into a new cell type through activation of different signalling pathways and transcriptional factors [248]. Recent studies have suggested employing conversion of human fibroblasts into neurons to study the neurodegenerative changes and observe what is happening inside the brain without intervention [249]. The direct reprogramming of fibroblasts includes direct conversion from one differentiated cell type to another different cell type without a reversion to iPSCs. In recent years, this novel approach has been demonstrated to be a fast and more efficient method to generate functional induced neurons (iNs) [250] and chemically induced neural stem cells [251].

In this context, a large body of evidence has shown that fibroblasts can be converted into GABAergic [35, 252], glutamatergic [35], and dopaminergic neurons [253] that represent the neuronal populations affected in AD, PD, HD, and ALS [21, 254–256]. Furthermore, other studies have successfully reported the conversion of human fibroblasts into neurons [238]. Both purified and recombinant proteins and chemical compounds have been identified to improve the direct programming into neurons [250, 257]. First, based on neural differentiation protocols, inhibition of the TGF/ALK/SMAD signalling pathway by application of recombinant Noggin and small-molecule ALK inhibitors, GSK-3β inhibitors, and cAMP/forskolin, has been identified to enhance neuronal conversion, improving purity and efficiency [258]. In this study, Pfisterer and colleagues demonstrated that all these compounds were necessary for effective neuronal reprogramming on day 12, showing neuronal purification rate of over 50% and expression of microtubule-associated protein 2 (MAP2) as a neuronal marker [258]. More importantly, fibroblasts converted into neurons display neuronal markers such as β-tubulin, MAP2, and glutamatergic transporter (GLUT1) [35]. Also, it has been shown that additional pathway modulations, including SIRT1 activation and HDAC inhibition, can boost the efficacy and maturity of the neuronal population obtained with the technique [258]. More importantly, the fibroblast-derived neuronal cells show intact neurophysiological properties such as voltage-channel currents, excitatory postsynaptic currents, and functional properties with the opening of sodium (Na^+^) and potassium (K^+^) channels during neuronal depolarization [259].

Similarly, other studies showed that chemical compounds could efficiently reprogram human fibroblasts to neurons [35]. These compounds are composed of two independent groups of molecules: combination A (CA) and combination B (CB) groups, which form a neuronal induction medium [35]. The CA group, which initiates neuronal transformation, consists of CHIR99021, LDN193189, RG108, and dorsomorphine (used as an essential chemical neuron-induction combination and inhibiting the fibroblasts signaling pathway), P7C3-A20, A83-01 and ISX9. The CB group, which maintains cell survival and enhances the reprogramming efficiency, consists of phosphocholine, Y27632, DAPT, PD0325901, A83-01, purmorphamine, and P7C3-A20. During the stepwise addition of CA and CB  at 0 (CA), 3 (CB), 6 (CA), 9 (CB), and 12 (maturation stage) days of culture of human fibroblasts, a neuronal conversion was observed, with a reprogramming efficiency of 82.1% ± 1.6% at 14 days [35]. However, the neuronal conversion efficiency on day 21 was diminished, probably due to the toxicity of these compounds induced by caspase-3 activation [35]. In addition, this study showed positive cells for neuronal proteins such as TUJ1, doublecortin, MAP2, and tau, indicating mature neurophysiological characteristics from day 14 [35].

Also, Dai and collaborators showed efficient conversion of skin fibroblasts into neurons from donors aged 6 months to 55 years [260]. The skin fibroblasts were cultured with high-glucose DMEM supplemented with a neuronal cocktail which contained inhibitors of TGF-β, bone morphogenic protein, GDK-3β, MEK-ERK, Pifithrin-α (inhibitor of P53), and forskolin, for 3 weeks [260]. Interestingly, after 3 weeks of treatment, the cells showed expression of neuronal markers such as βIII-tubulin and MAP2 along with arborized neurites, with cell proportions increased from 3 weeks (88.2% ± 3.9%) to 4 weeks of exposure (89.2% ± 2.1%) [260]. Interestingly, the neuronal cells reprogrammed from skin fibroblasts after 3-week exposure showed expression of markers of glutamatergic (VGlut1), GABAergic (GABA), and dopaminergic (tyrosine hydroxylase) neurons [260]. On the other hand, motor neurons have also been reprogrammed from skin fibroblasts obtained from human adults using the transcriptional factor POU5F1 (involved in the plasticity of somatic cells) and LHX3 overexpression (motor neuron specification factor) [261]. Furthermore, reprogrammed motor neurons show expression of ISL1, HB9, NKX6.1, and LHX3 protein markers, which are highly expressed during motor neuron conversion and reprogramming of the neuronal lineage [261]. In this study, the reprogrammed motor neurons show depolarization currents and spontaneous firing at the resting membrane potential [261].

More interestingly, skin fibroblasts have been used to model and study AD, PD, HD, ALS, and other neurodegenerative disorders [262]. For example, neurons reprogrammed from skin fibroblasts obtained from AD patients show high levels of the main hallmarks of this neuropathology, including Aβ (1–40 and 1–42) and hyperphosphorylated tau, compared to reprogrammed neurons obtained from healthy patients [250]. In addition, a recent study suggested that fibroblasts from senescent patients present with high APP mRNA expression [263]. Therefore, the use of skin fibroblasts could be considered as an exciting model to study the mechanisms of AD. More importantly, fibroblasts have also been used to study the progression of mitochondrial health in AD [25, 29]. Mitochondrial function is essential for neuronal function and communication [264], and failure of these organelles is directly linked to the pathogenesis of AD, PD, HD, and ALS. From this point, direct conversion technologies have increased rapidly, where they are seen as a helpful approach to generating different cell types from human patients and donors for disease modelling, potential regeneration [207, 208], drug development, and offering advantage of safety [243, 265], or even for cell replacement strategies [266].

Interestingly, studies using the iNs method to convert fibroblasts into neurons showed that these cells could survive for at least two months and show potent electrophysiological activity when co-cultured with astrocytes [35]. Notably, after transplanting the reprogramed neurons into the mouse brain, the cells could successfully survive in vivo and integrate into the resident circuits [35].

Currently, further studies are urgently needed to improve iNs generation from adult human fibroblasts since the low efficiency represents an obstacle for further applications.

One of the major limitations for direct conversion is that the starting material is finite, and the conversion process does not involve the expandable stem cells. As a result, the iNs cell numbers are low, and the ability to scale up the system is poor, especially for large-scale studies [243, 265]. In addition, it is challenging to effectively deliver small molecules or transcription factors across the brain-blood barrier to the affected brain [35, 243, 265]. Another issue is that the final converted neurons are mostly glutamatergic [35], although some studies indicate that dopaminergic neurons can also be achieved [258].

Significantly, many physical factors can improve the reprogramming efficiency, indicating strong application prospects. The physical factors mainly have two advantages in the reprogramming of fibroblasts: (1) there is no need to apply transcription factors by viruses or other routes, thus preventing associated toxic and side effects; and (2) there is no direct manipulation of genetic materials [241, 242] (Table 1). Hence, the physical factor-mediated reprogramming is safer and has better security and applicability, making it a valuable strategy to establish and study different disease models and for clinical treatment of patients.

## Mitochondrial dysfunction in fibroblasts obtained from AD, PD, HD, and ALS patients

**Fig. 3:**
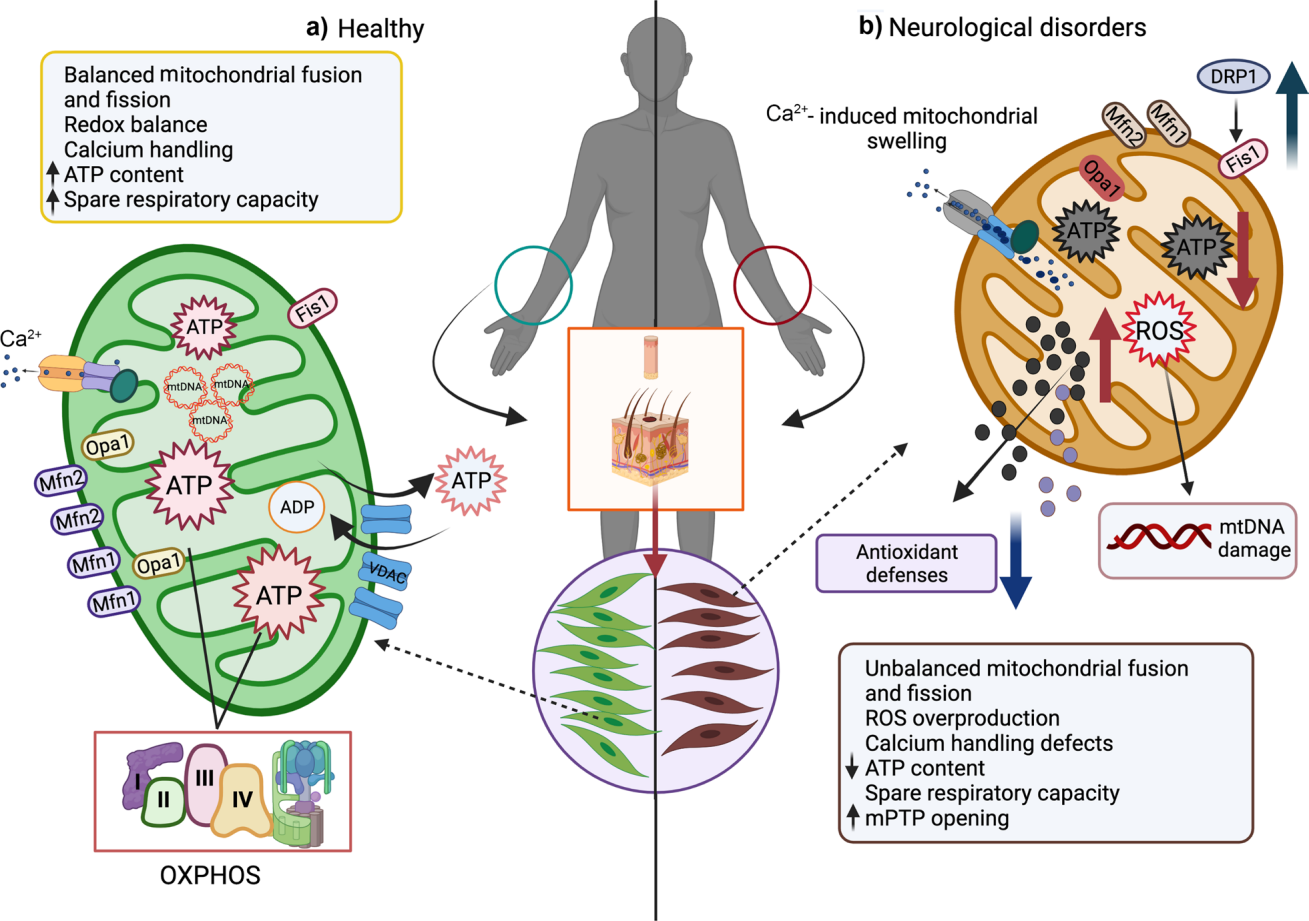
Mitochondrial damage is present in fibroblasts obtained from patients with NDs. Current evidence shows that skin fibroblasts display similar mitochondrial features as in neuronal cells, either in normal or pathological conditions. In non-pathological conditions, skin fibroblasts show healthy mitochondria highlighted by OXPHOS activity, high ATP production, calcium homeostasis, redox balance, and mitochondrial dynamics balance (**a**). Importantly, mitochondrial failure has been studied in brains of patients with NDs, and also reported in skin fibroblasts from ND patients. In this context, skin fibroblasts obtained from ND patients show mitochondrial swelling through mPTP opening, reduced ATP levels, oxidative stress, decreased antioxidant defenses, redox imbalance, mtDNA damage, and accumulation of fragmented mitochondria (**b**). These mitochondrial features are critical to synaptic function, and any abnormalities in these processes would contribute to neurodegeneration

Accumulative studies have shown several signs of mitochondrial dysfunction in cortical samples from post-mortem AD, PD, HD, and ALS patients [267–269]. Also, cultured fibroblasts obtained from patients with different NDs have shown abnormal mitochondrial functions [28, 29, 171, 245, 270–272] (Fig. 3). For example, fibroblasts from sporadic and familial AD patients show a reduced Δψm (35%) and abnormal mitochondrial respiration where the spare respiratory capacity was reduced (34%) compared to controls [273]. These mitochondrial deficiencies are accompanied by mitochondrial fragmentation and accumulation around the perinuclear area [273].

Furthermore, studies in fibroblasts from patients with PD-associated *PARK2* mutations show a significant decrease in Δψm, together with significant mitochondrial proteomic alterations [274]. Studies in human fibroblasts showed that OXPHOS inhibition is also a key feature of mitochondrial dysfunction [275]. Also, these studies show that human skin fibroblasts present with defects in OXPHOS, reduced oxygen consumption, decreased ATP production, and mitochondrial fragmentation due to mutations in mtDNA [275].

In the next section, we will discuss several findings of mitochondrial dysfunction in fibroblasts obtained from patients with AD, PD, HD, and ALS, which can be considered as an essential tool to evaluate mitochondrial failure in these patient brains.

### AD

As mentioned in this review, several studies have demonstrated the presence of mitochondrial dysfunction in the brains of AD patients [76, 276–278] and that this could be associated with the impairment of synaptic transmission and cognitive decline present during this disease [279, 280]. Furthermore, as was described before, abnormal mitochondrial dynamics, bioenergetics impairment, and oxidative damage are observed in AD [20, 93, 102]. Interestingly, our studies and others showed that mitochondrial damage is observed in fibroblasts obtained from patients diagnosed with sAD [29, 39, 275, 281]. Complementary studies by Gray and Quinn demonstrated that skin fibroblasts from AD patients who had mutations in PSEN1 which represents the most common cause of fAD, showed significant changes in mitochondrial mass and number compared to age-matched fibroblasts [282]. Other findings revealed that skin fibroblasts from sAD patients showed a decrease in the mRNA levels of mitochondrial dynamics regulators, including Mfn2, OPA1, and DRP1 [283]. However, other studies showed that mitochondrial fission is affected in fibroblasts from sAD patients, showing an increase in the interaction of Dynamin-like protein 1 and Fis1 compared with healthy controls [284]. Complementarily, AD fibroblasts show a significant decrease in mitochondrial length, presenting a shorter and fragmented morphology, suggesting evident mitochondrial defects in these cells compared to age-matched cells [29].

Interestingly, these results are associated with irregular proteins that regulate mitochondrial dynamics, as observed in sAD patients [29]. In this context, the OPA1 protein is proteolytically cleaved by metalloproteases OMA1 and YMEL1, generating two isoforms (long and short OPA1) [285], and it is thought that OMA1 processing increases mitochondrial fission [286]. Our previous studies showed significant decreases in the 71-kDa band of Mfn1 and the long isoform of OPA1, as well as a notable increase in the short isoform of OPA1, in AD and PD [29]. In addition, other studies evaluated the mitochondrial recycling process by autophagy in two different human cell models of fAD-associated *PSEN1* A246E mutation: unmodified skin fibroblasts and iPSC-derived neurons. The skin fibroblasts harbouring fAD mutations showed dysregulation in the degradation phase of autophagy correlating with lysosomal anomalies, leading to the accumulation of dysfunctional mitochondria [287]. Consistent results were obtained in neurons derived from AD patients’ iPSCs that presented the same mutation [287].

Interestingly, these authors previously described similar mitophagy failure in fibroblasts and brain samples of sAD patients [287]. However, the etiology of the defect is not the same. In sAD, the deficit is due to insufficient labelling of mitochondria to be degraded by mitophagy; on the contrary, in fAD patients, mitochondria are correctly tagged for recycling, but there is a defective degradation by the autophagy process [287]. Importantly, these studies demonstrated that the fAD-fibroblasts retain mitophagy pathology found in fAD neurons and fAD patient brains, further validating their use as models for AD study.

Furthermore, other studies evaluated the effect of oxidative stress in fibroblasts of sAD patients [288]. The authors compared the gene expression profile of normal human fibroblasts exposed to oxidative stress with the gene expression profile of sAD-patient derived fibroblast cell lines [288]. Using cDNA microarray, they determined the expression of > 14,000 genes, finding 1017 chronically down-regulated genes in AD fibroblasts [288].

Abnormalities in mitochondrial bioenergetics have also been observed in sAD fibroblasts [29, 39, 289]. Decreased ATP levels, oxidative stress, and defects in calcium handling have been widely documented [29, 39, 290, 291] (Fig. 3). Furthermore, ROS overproduction has been widely considered a vital contributor to AD [292]. In this context, skin fibroblasts from fAD patients show high ROS levels and oxidative damage represented by an increase of 4-hydroxynonenal, which indicates lipid peroxidation, and an increase in protein carbonyl content compared to control cells [293]. Recently, our studies demonstrated that sAD fibroblasts show a significant ROS accumulation, mitochondrial depolarization, reduced ATP production, and mitochondrial calcium handling defects [29, 39]. More importantly, we observed a relevant role of mPTP in the mitochondrial dysfunction present in AD fibroblasts [39]. Briefly, mPTP has been intensely implicated in mitochondrial dysfunction, leading to calcium mishandling, increased ROS generation, and decreased ATP production, which are observed in AD pathophysiology [294–296].

Interestingly, blocking the mPTP opening with cyclosporine A prevents ROS increase, induces a decrease in superoxide levels and reduces mitochondrial calcium dysregulation in AD fibroblasts [39]. Additionally, mtDNA damage is considered in studies as a risk factor for AD, leading to increased mitochondrial fractionation and decreased mitochondrial content, even affecting cellular DNA and triggering neuronal apoptosis [297–299]. In this context, fibroblasts obtained from 129 sAD patients and 40 fAD patients show mutations in mtDNA, identifying nucleotide modifications of NADH dehydrogenase (3337–3340) which could contribute to the mitochondrial dysfunction observed during AD [300]. Additionally, these negative changes in mtDNA affect the expression of genes related to the mitochondrial complex in ETC [298]. Accumulative studies showed decreases of ETC activity and expression of mitochondrial respiratory chain complexes I-V, which reduce the respiratory capacity in AD fibroblasts [282, 301, 302].

Furthermore, Drabik and co-workers studied fibroblasts obtained from sAD patients, and they demonstrated alterations in Ca^2+^ homeostasis, increased mitochondrial ROS production, and altered mitophagy and autophagy processes, highlighting the presence of cellular stress in AD fibroblasts [303]. The authors discussed that the effects concerning intracellular Ca^2+^ levels in AD fibroblasts were not consistent. In fAD models, increased cytosolic Ca^2+^ is frequently observed; however, a decrease has also been reported depending on the type of mutation causing fAD [304]. These discrepancies between fAD and sAD models concerning calcium homeostasis underline the need for proper experimental tools and setups for sAD investigation.

Notably, previous findings by Bell and collaborators also showed defects of mitochondrial bioenergetics, reduced mitochondrial membrane potential, reduced oxygen consumption, and diminished mitochondrial respiratory capacity and ATP production in both sAD and fAD fibroblasts [273]. These alterations were accompanied by morphological changes such as increased mitochondrial fragmentation and reduced mitochondrial content [273]. However, these studies did not detect changes in mitochondrial mass, excess ROS production, transmembrane instability, or DNA deletions [305]. Altogether, skin fibroblasts obtained from patients with sporadic or familial AD display mitochondrial injury observed in several AD models and brain samples of post-mortem AD patients.

### PD

As mentioned above, the association between PD and mitochondrial impairment has been strongly suggested [306]. Skin fibroblasts from sPD patient donors show aberrant changes in mitochondrial morphology [28]. For example, studies by Antony and collaborators showed that sPD fibroblasts  have reduced mitochondrial perimeter and increased mitochondrial fragmentation, which lead to a significant reduction of the mitochondrial network [28]. Furthermore, they showed that abnormal mitochondrial morphology correlates with the abnormal and slowed growth of fibroblasts from sPD patients compared to age-matched control fibroblasts [28]. This study also observed mitochondrial hyperpolarization, which indicates overcompensation for mitochondrial stress in sPD fibroblasts [28]. In addition, the authors discuss that these findings are contradictory to those found in familial forms of parkinsonism, particularly LRRK2 G2019S18 and several Parkin mutants, where mitochondrial depolarization has been observed [28].

Additional reports in sPD patients showed abnormalities in fibroblast growth (without affecting cell viability) associated with mitochondrial dysfunction [307] (Fig. 3). Consequently, sPD fibroblasts showed a smaller area and a reduced perimeter associated with susceptibility to oxidative stress induced by ultraviolet exposure (environmental stressor) [307]. Interestingly, defects in mitophagy have been found in fPD fibroblasts obtained from patients carrying two mutated *Parkin* or *PINK1* alleles [308]. Mitophagy is a crucial process where degradation of mitochondria occurs via autophagy, thereby maintaining mitochondrial homeostasis [309]. This process is commanded by two pivotal proteins called PINK and Parkin, which initiate mitochondrial degradation [310]. However, mitophagy failure has been observed in PD with mutation or loss of PINK1 and Parkin, reducing the degradation of damaged mitochondria, leading to their accumulation and neuronal dysfunction [311]. It is widely understood that PINK1 accumulates in damaged mitochondria, recruits Parkin to ubiquitinate the mitofusins 1 and 2, and promotes fissions, ultimately activating the mitophagy process [312]. However, it has been reported that PINK1 and Parkin mutations affect the mitophagy process in fPD fibroblasts [308]. Also, fibroblasts from fPD patients with *Parkin* mutation are shown to be more susceptible to mitochondrial morphological changes, inducing mitochondrial fragmentation and inhibiting mitochondrial fusion [313]. In addition, loss of PINK1 function has been associated with mitochondrial impairment present in PD [314]. For example, studies in fPD skin fibroblasts with functional loss of PINK1 protein showed alterations of mitochondrial morphology, leading to accumulation of fragmented mitochondria [315]. Parkin is recruited to damaged mitochondria, where it ubiquitinates Mfn1 and Mfn2 [316]. A study reported that control fibroblasts treated with valinomycin (apoptosis inducer) and CCCP (uncoupler of OXPHOS) show Mfn2 deficiency, leading to mitochondrial fragmentation and therefore to the elimination of damaged mitochondria [308].

In contrast, in fPD fibroblasts, Mfn2 levels remain unchanged after induction of mitochondrial stress, suggesting that the mitophagy process is altered [308]. These events probably occur since the PINK mutation present in PD fibroblasts has been found to prevent Parkin translocation into stressed mitochondria, inhibiting the ubiquitination of both mitofusins (Mfn1/2) [317]. This could explain the accumulation of impaired mitochondria and the deficit of mitochondrial clearance observed in fPD fibroblasts [318]. Recent data also indicate that autophagy is regulated by protein acetylation mediated by histone acetyltransferase (HAT) and histone deacetylase (HDAC) activities [318]. Furthermore, protein modifications like acetylation have been involved in PD and sPD pathogeneses [318]. In this context, fibroblasts from PD patients with the G2019S LRRK2 mutation show elevated mitophagy due to the activation of class III HDACs, while sPD fibroblasts exhibit downregulation of damaged mitochondrial clearance [318].

Although fibroblasts display a lower bioenergetic burden than neurons, mitochondrial changes such as reduced pyruvate oxidation and inhibition of complexes I, IV, and V have been reported in sPD-derived fibroblasts, confirming that energy supply is decreased in these cells [319]. Thus, the impairment of mitochondrial bioenergetics has been widely documented in PD, evidencing several abnormalities in ETC activity and ROS management [320–322].

Mitochondrial respiration deficiencies observed in PD neuronal models are also present in fibroblasts from fPD or sPD patients [25]. For example, the activity of the mitochondrial complex I is significantly inhibited in the brain cortex of PD patients [323, 324]. These changes have also been confirmed in skin fibroblasts of PD patients, which show low activity of mitochondrial complex I and reduced mitochondrial mass [325]. Further studies in fibroblasts have found that the expression of complex V (ATP-synthase) is reduced in the substantia nigra of post-mortem PD patients, which could consequently reduce this activity [271, 326].

Lastly, the accumulation of α-synuclein represents an important pathological hallmark present in PD, which leads to mitochondrial failure in dopaminergic neurons [327, 328]. PD fibroblasts with α-synuclein overexpression show significant defects in mitochondrial function, higher susceptibility to oxidative damage, decreased ΔΨm and ATP synthesis, and deficits of complex I activity, compared to age-matched control fibroblasts [329]. Studies in fibroblasts with mutations of PARK2, which cause juvenile autosomal recessive PD, have also revealed mitochondrial failure [330]. These fibroblasts show dissipation on ΔΨ and severe deficits in ATP production; however, they do not show fragmented mitochondria, possibly due to the defects in the mitophagy process, resulting in accumulation of damaged mitochondria not targeted to mitophagy [330]. The Parkin mutant in PD fibroblasts also triggers a cascade of mitochondrial damage by increasing the levels of oxidized proteins, impairing respiratory complexes II, III and IV, and increasing mitochondrial mass compared to control cells [331]. Therefore, PD skin fibroblasts display abnormal mitochondrial characteristics observed in brain samples, animal models, and cellular PD models, suggesting that the patient-derived human dermal fibroblasts are a powerful model for studying PD.

### HD

Mitochondrial damage has also been observed in fibroblasts from patients diagnosed with HD [40, 332]. An electron microscopic study in HD skin fibroblasts showed increased mitochondrial size with an active swelling process with disorganized crests and an altered matrix [333]. Similarly, other studies demonstrated a significant reduction in mitochondrial number and density in HD skin fibroblasts (between 20% and 60%), associated with neuronal atrophy observed in HD [171]. These effects can be explained, since studies in HD skin fibroblasts have shown a co-localization between mutant HTT and DRP1 [334]. Furthermore, these HD cells show fragmented mitochondria with small and round shapes compared to control fibroblasts, and alterations in organelle transport, reducing both anterograde and retrograde mitochondrial movement [334].

On the other hand, several studies have shown defects of mitochondrial bioenergetics in fibroblasts from HD patients [40, 332]. Mitochondrial-related ROS increase and oxidative damage have been reported in skin fibroblasts from HD patients [40]. Significantly lower activities of the antioxidant-related enzyme catalase have been found in skin fibroblasts of HD patients compared to controls (Fig. 3) [40]. Also, HD skin fibroblasts have been shown to have slower growth and proliferative behaviour than age-matched control fibroblasts, which is linked to mitochondrial damage [171]. In this condition, work from Jędrak et al. showed that ATP levels are reduced in HD skin fibroblasts in the presence of glucose or galactose, together with an increased cytosolic ROS level as well as O_2_^−^; however, the Δψ and the expression of all ETC complexes (I–IV) did not show changes in HD skin fibroblasts [332]. Complementarily, activities of antioxidant enzymes were also measured, revealing an increase in superoxide dismutase 2 (SOD2), which, as proposed by the authors, might be due to the high ROS levels. At the same time, activities of catalase, glutathione peroxidase, and reductase were reduced in HD skin fibroblasts [332]. Notably, symptomatic HD patients have decreased levels of mitochondrial complexes II, III, and IV in different brain regions [178, 179, 335].

In addition to these findings, mtDNA damage has also been observed in HD fibroblasts, which show a reduced number of mtDNA copies, probably due to the inhibition of mitochondrial fusion and increased fragmentation [336]. Altogether, fibroblasts derived from HD patients have negatively altered mitochondrial phenotypes leading to cellular susceptibility to oxidative stress [337].

### ALS

Fibroblasts from ALS patients have also shown aberrant changes in mitochondrial quality control, characterized by abnormal changes in dynamics, degradation, and biogenesis [338]. ALS fibroblasts exhibit mitochondrial fragmentation caused by an altered balance of fission and fusion processes shown with increased Fis1 expression, and decreased mitochondrial number, presenting disorganized crests [338]. Cristae organizing system in ALS fibroblasts also shows a loss of integrity of mitochondria crests, associated with increased mitochondrial fission and decreased LC3 and ps6, enhancing the ALS severity [338, 339].

Regarding defects in mitochondrial bioenergetics in ALS fibroblasts, OXPHOS activity, loss of antioxidant activity, ROS overproduction, and redox imbalance have been observed [196, 340]. In addition, aberrant changes in energy metabolism have also been found in ALS fibroblasts [237]. For example, studies from Allen et al. reported that fibroblasts obtained from ALS patients with SOD1 mutation showed a significant reduction in mitochondrial oxygen consumption and mitochondrial respiratory capacity compared with control fibroblasts [237]. Moreover, the authors showed that these energetic changes observed in mitochondria of ALS fibroblasts are exacerbated as the disease progresses [341].

Dysregulation of the OXPHOS activity has been an evident hallmark in ALS fibroblasts, including a time-dependent decrease in complex I activity along with a deficiency in ATP synthesis, which is associated with response to the SOD1 mutation [341, 342]. Furthermore, another study in ALS fibroblasts also identified changes in OXPHOS activity, promoting evidence of decreased ADP content and low ATP production compared to control fibroblasts [343]. Also, these authors showed that OXPHOS uncoupling led to a decrease in the mitochondrial electrochemical gradient of protons associated with an increased proton permeability and reduced respiratory rate, affecting energy metabolism [343]. Furthermore, other researchers have reported that ΔΨm is increased in ALS fibroblasts due to a possible compensatory response to energy deficit [26] (Fig. 3). Also, oxidative stress is widely evidenced in ALS [344–346]. Studies suggest that the oxidative damage occurs through the ERK1/2 pathway in ALS fibroblasts [41] which regulates the cellular response to stress [347], while inhibition of the ERK1/2 pathway with PD980059 reduces oxidative damage and increases the expression of antioxidant enzyme glutathione (GSH) [41].

## Conclusions

This review discusses evidence that fibroblast cells present similar mitochondrial deficiencies to those in neuronal cells, mouse models, and brain samples from AD, PD, HD, and ALS patients. Therefore, fibroblasts are an excellent candidate to study the genesis and progression of these diseases. Interestingly, skin fibroblasts reflect metabolic changes observed in the brains of NDs, indicating the possible use of these cells as a diagnostic tool to study neurodegenerative changes present in NDs. Furthermore, the direct reprogramming of ND fibroblasts achieved by transgene-free or chemical-only approaches may serve as an alternative for safer strategies in generating neuronal cells to study mitochondrial dysfunction. Nevertheless, more research is needed to improve the efficiency and reduce the variations of induction processes. In this case, future studies are needed to boost iNs generation from adult human fibroblasts, considering the current low efficiency of the technique that hinders further applications. Future expectations involve the 3D culture that may represent an exciting tool and facilitate chemical reprogramming.

Notably, studies of mitochondrial function in skin fibroblasts in NDs can generate exciting information to propose other molecular targets, such as mPTP and the antioxidant Nrf2. Furthermore, as presented in this review, mitochondrial abnormalities in bioenergetics and dynamics may contribute to AD, PD, HD, and ALS, affecting synaptic transmission, leading to cognitive impairment observed in these diseases.

Finally, the use of ND fibroblasts would allow us to track the progression of mitochondrial dysfunction and make early interventions to reduce neurodegenerative changes observed in these NDs. Finally, these novel approaches may provide insights into the future cell replacement therapies to treat mitochondrial dysfunction in NDs.

## Data Availability

References' information used in this manuscript will be available on demand.

## Abbreviations

AD: Alzheimer's disease
ALS: Amyotrophic lateral sclerosis
APP: Amyloid precursor protein
DRP1: Dynamin related protein 1
ETC: Electron transport chain
fAD: Familiar Alzheimer’s disease
fPD: Familiar Parkinson’s disease
HD: Huntington’s disease
HDAC: Histone deacetylase
hiPSC: Human induced pluripotent stem cells
iNs: Induced neurons
iPSC: Induced pluripotent stem cells
Mfn1/2: Mitofusin 1 and 2
mHtt: Mutant huntingtin
mPTP: Mitochondrial permeability transition pore
mtDNA: Mitochondrial-DNA
NDs: Neurological disorders
NFTs: Neurofibrillary tangles
NMDA: *N*-methyl-*D*-aspartate
OPA1: Optic atrophy protein 1
OXPHOS: Oxidative phosphorylation
PD: Parkinson’s disease
ROS: Reactive oxygen species
sAD: Sporadic Alzheimer: s disease
SOD: Superoxide dismutase
sPD: Sporadic Parkinson’s disease
VDAC: Voltage-dependent anion-selective channel
Δψm: Mitochondrial membrane potential
